# The Incremental Prognostic Value of E/(e’×s’) Ratio in Non-ST-Segment Elevated Acute Coronary Syndrome

**DOI:** 10.3390/diagnostics11081337

**Published:** 2021-07-26

**Authors:** Ioana Ionac, Mihai-Andrei Lazăr, Daniel Miron Brie, Constantin Erimescu, Radu Vînă, Cristian Mornoş

**Affiliations:** 1 Cardiology Department, “Victor Babes” University of Medicine and Pharmacy, 300041 Timisoara, Romania; ioana_ionac@yahoo.com (I.I.); mornoscristi@yahoo.com (C.M.); 2 Cardiology Department, Institute of Cardiovascular Diseases, 300310 Timisoara, Romania; brie_daniel@yahoo.com (D.M.B.); costelerimescu79@gmail.com (C.E.); 3 Viami Software, Viami Solution SRL, 011334 Bucharest, Romania; raduvina@gmail.com

**Keywords:** tissue Doppler imaging, mitral annulus velocities, prognostic, non-ST-segment elevated acute coronary syndrome, cardiac events

## Abstract

It has been shown that the E/(e’×s’) index, which associates a marker of diastolic function (E/e’, early transmitral/diastolic mitral annulus velocity ratio) and a parameter that explores LV systolic performance (s’, systolic mitral annulus velocity), is a good predictor of outcome in acute anterior myocardial infarction. There are no studies that have investigated the prognostic value of E/(e’×s’) in a non-ST-segment elevated acute coronary syndrome (NSTE-ACS) population. Echocardiography was performed in 307 consecutive hospitalized patients with NSTE-ACS and succesful percutaneous coronary intervention, before discharge and six weeks after. The primary endpoint consisted of cardiac death or readmission due to re-infarction or heart failure. During the follow-up period (25.4 ± 3 months), cardiac events occurred in 106 patients (34.5%). Receiver operating characteristic (ROC) analysis identified E/(e’×s’) at discharge as the best independent predictor of composite outcome. The optimal cut-off value was 1.63 (74% sensitivity, 67% specificity). By multivariate Cox regression analysis, E/(e’×s’) was the only independent predictor of cardiac events. Kaplan–Meier analysis identified that patients with an initial E/(e’×s’) > 1.63 that worsened after six weeks presented the worst prognosis regarding composite outcome, readmission, and cardiac death (all *p* < 0.001). In conclusion, in NSTE-ACS, E/(e’×s’) is a powerful predictor of clinical outcome, particularly if it is accompanied by worsening after 6-weeks.

## 1. Introduction

Patients admitted to hospital because of an acute coronary syndrome (ACS) constitute a heterogeneous population with a varying risk of future cardiac events [1,2]. Non-ST-segment elevated acute coronary syndrome (NSTE-ACS) is a significant contributor to both morbidity and mortality, accounting for almost half of all deaths related to cardiovascular disease [3,4]. Risk stratification is important to identify patients who may benefit from an intensified treatment strategy [1,4] and it helps prevent unnecessary re-admissions to the hospital [4]. The prognosis of patients with NSTE-ACS is related to the occurrence and extent of myocardial necrosis and left ventricular (LV) remodeling [5]. Prior to the clinical symptoms of heart failure (HF) becoming clear, patients can develop asymptomatic LV dysfunction caused by structural or functional cardiac abnormalities [2]. Ischemia with subsequent impairment of myocardial contractility and myocardial damage are related to increased LV filling pressure [6].

Many parameters for the indirect assessment of LV filling pressures have been used to assess cardiac outcomes in patients with coronary artery disease. Recent studies have shown tissue Doppler imaging (TDI) to be a strong predictor of adverse outcomes in several cardiac diseases. A significant decrease of systolic mitral annulus velocity (s’) and/or early diastolic mitral annulus velocity (e’) has been observed in patients with ischemic heart disease, even if cut-off values for peak systolic and diastolic velocities which detect significant coronary artery stenosis are still unclear [1,6]. Several authors have identified that the e’ velocity and the ratio of early transmitral flow velocity to early diastolic mitral annulus velocity (E/e’) are highly predictive of adverse events after acute myocardial infarction [4,6,7,8,9,10]. The s’ wave can be used to quantify the entity of regional motion impairment [6,10]. This velocity is related to the longitudinally orientated myocardial fibers located in the sub-endocardium, which is known to be most susceptible to ischemia [2]. Agarwal et al. suggest that s’ velocity may be a more powerful predictor of outcome than LV ejection fraction (LVEF), as it may reflect subclinical LV systolic dysfunction [6].

The idea of a complex marker that incorporates several tissue Doppler parameters was used by several study teams. Our group proposed a new TDI index, E/(e’×s’), that associates a marker of diastolic function (E/e’) and a parameter that explores LV systolic performance (s’). We demonstrated the utility of this index to assess the LV filling pressure in a heterogeneous population of cardiac patients, regardless of LVEF [11] and to improve the prediction of cardiac events in patients with HF [12]. By analyzing patients with acute anterior myocardial infarction, Kenar Tiryakioglu et al. reported that the E/(e’×s’) ratio can strongly predict LV remodeling after a 6-month follow-up [13]. Unfortunately, there are no studies that have investigated the prognostic value of E/(e’×s’) in an NSTE-ACS population. This is what we aim to examine: the association between E/(e’×s’) ratio and the cardiac outcome, and the value of E/(e’×s’) worsening during follow-up.

## 2. Materials and Methods

### 2.1. Study Population

The Cardiology Department of Timisoara Institute of Cardiovascular Diseases is an invasive hub for 15 non-invasive cardiology departments. In the period November 2017 to January 2019, 2758 patients were admitted to the department for percutaneous coronary intervention (PCI). These patients were all included in a clinical registry. We analyzed prospectively 439 consecutive patients with NSTE-ACS and successful PCI, in sinus rhythm, hospitalized in our clinic. Successful PCI was defined as residual stenosis ≤20%; non-culprit intervention was performed during the same hospitalization. Patients with inadequate echocardiographic images, previous myocardial infarction, open-chest surgery, cardiac pacemaker/defibrillator, significant valvular heart disease, renal failure (serum creatinine >1.3 mg/dL), and non-cardiac disease with a life expectancy <1 year were excluded from the study group. The remaining 307 patients formed our study population. The study was conducted according to the guidelines of the Declaration of Helsinki and approved by the Institutional Ethics Committee of Institute of Cardiovascular Diseases Timisoara (protocol code 1658 and 28 March 2014). Informed consent was obtained from all participants.

### 2.2. Clinical Variables Recorded

The following clinical variables were recorded and included in the prognostic model: age, sex, mean arterial pressures, heart rate, body mass index, peak high sensitivity cardiac troponin I level (hs-cTnI), and N-terminal pro-brain natriuretic peptide (NTproBNP) levels. Prescription of the main therapeutic classes was also recorded.

For study purposes, five cardiovascular risk factors were considered as follows: hypertension (systolic blood pressure >140 mmHg, diastolic blood pressure >90 mm Hg, or in drug treatment), cardiovascular disease heredity, smoking (≥1 cigarette/day, cessation of smoking <10 years previously was still considered as smoking), diabetes mellitus (fasting glycemia >126 mg/dL or in drug treatment), and hypercholesterolemia (>200 mg/dL or in drug treatment).

### 2.3. Echocardiography

Echocardiography was performed after PCI, at hospital discharge, using a Vivid 9 General Electric, Milwaukee, WI system, USA. Left atrial volume (LAV) and indexed LAV to the body surface area (LAVI) were determined according to current guidelines [14]. LVEF was calculated from apical two- and four-chamber views using a modified Simpson’s rule [14]. The regurgitant orifice area (ROA) and the regurgitant volume (RV) of mitral regurgitation were determined [15]. Transmitral flow patterns were recorded from apical four-chamber windows with a 3–5 mm pulsed-sample Doppler volume placed between mitral valve tips. Peak E and late transmitral flow (A) were measured for five consecutive cardiac cycles during end-expiratory apnoea, and the results were averaged [8]. Systolic pulmonary artery pressure (SPAP) was estimated from peak velocity of tricuspid regurgitation.

The TDI program was set in pulsed-wave Doppler mode. In the apical four-chamber view, a 4–5 mm sample volume was positioned sequentially at the lateral and septal corner of the mitral annulus [8]. Peak e’ and s’ were recorded for five consecutive cardiac cycles during end-expiratory apnoea, and the results were averaged. E/e’ and E/(e’×s’) were calculated by using average velocities of septal and lateral site. TDI measurements were repeated six weeks after hospital discharge (42 ± 5 days). E/(e’×s’) worsening was defined as a value greater to the previous value determined before discharge.

All measurements were performed by an experienced echocardiographer. The inter- and intra-observer variability for E/e’, s’ and E/(e’×s’) were examined. Measurements were performed in a group of 30 randomly selected subjects by one observer at two separate times and by two investigators who were unaware of the other’s measurements and of the study time point.

### 2.4. Clinical Outcome

Patients were followed for ≥24 months. The primary event consisted of cardiac death or hospital readmission for HF or re-infarction. Cardiac death was defined as either a death directly related to cardiac disease, mainly congestive HF, re-infarction, or sudden death. The follow-up information was obtained from electronic medical files or by telephone contact with the patients or their family members.

### 2.5. Statistical Analysis

Data were expressed as mean ± standard deviation (SD) for continuous variables and as proportions for categorical variables. Continuous variables were compared between groups using an unpaired *t*-test (variables with normal distribution) or Mann–Whitney U test (non-normally distributed variables). Proportions were compared using χ^2^ test and Fischer’s exact test. Receiver operating characteristic (ROC) curves for predicting cardiac events were determined for different parameters, and area under the ROC curves (AUC) were compared. Patients who died of non-cardiac causes were censored at the time of death.

Uni- and multivariable Cox regression models were created to correlate clinical, biological, and echocardiographic findings to the primary endpoint. For multivariable Cox regressions, three models with an incremental number of confounders were constructed. Model 1 included either E/(e’×s’), s’, or E/e’ and the following confounders: age, gender, mean blood pressure, heart rate, body mass index, prior coronary artery disease, family history of cardiovascular disease, diabetes mellitus, current smoker, hypercholesterolemia, and hypertension. In Model 2, we also included variables obtained from the laboratory (NTproBNP level, hs-cTnI), coronary angiography (culprit lesions, multivessel disease), and ACS category. Model 3 additionally included all significant echocardiographic parameters (LVEF, SPAP, LAV, LAVI, E, E/A, and e’). Harrell’s c-statistics was calculated from univariable Cox regression for all measures included in the multivariable Cox regression to compare the predictive potential of baseline predictors. The cardiac event-free survival rates were calculated using the Kaplan–Meier analysis, and the event rates were compared using the log-rank test. A *p*-value < 0.05 was considered significant. Intra-observer variability and inter-observer variability for E/e’, s’ and E/(e’×s’) were measured by Bland–Altman analysis, and intraclass correlation coefficients. We used STATA Statistics/Data analysis, MP 12.0 (StataCorp, College Station, TX, USA) as statistical software.

## 3. Results

In the period of November 2017 to January 2019, 307 consecutive patients hospitalized for NSTE-ACS who underwent successful PCI were included in this prospective study. All patients received standard management according to the institutional recommendation. The mean age of our study sample was 61 ± 12 years and 70.7% were males (217 patients). In this study, no patient was lost to follow-up. During the follow-up period (25.4 ± 3 months), cardiac events occurred in 106 patients (34.5%). The first cardiac event was represented by cardiac death in seven patients (2.28%) and readmission in 99 patients (32.2%). Of our patients, 72 (23.4%) were hospitalized for heart failure, and 17 (5.5%) had a non-fatal re-infarction. During the follow-up, cardiac death occurred in 22 patients (7.16%).

### 3.1. Patients’ Characteristics

Patients’ baseline characteristics are presented in Table 1. Patients with cardiac events had higher body mass index, incidence of non-ST elevation myocardial infarction, NTproBNP and hs-cTnI levels, SPAP, E, E/A, E/e’ ratio and E/(e’×s’), larger LAV, lower index LV end-systolic volume, LVEF, and s’ velocities. In addition, there was no difference regarding the distribution of age, gender, previous coronary artery disease, heart rate, and mean arterial pressure during the baseline echocardiogram, cardiovascular risk factors (diabetes, smoking, hypertension, heredity, dyslipidemia), medication (regarding beta blocker, calcium blocker, angiotensin converting enzyme inhibitor/angiotensin receptor antagonist, nitrates, statin, and diuretics), localization of culprit lesion, LAVI and LV end-diastolic volume index, E-deceleration time, ORA, RV, and e’. Mean E/(e’×s’) at discharge was 1.61 ± 1.12 in patients without events, while it was 2.22 ± 1.03 in the rest (*p* < 0.001).

### 3.2. ROC Curves to Predict Cardiac Events

**Figure 1:**
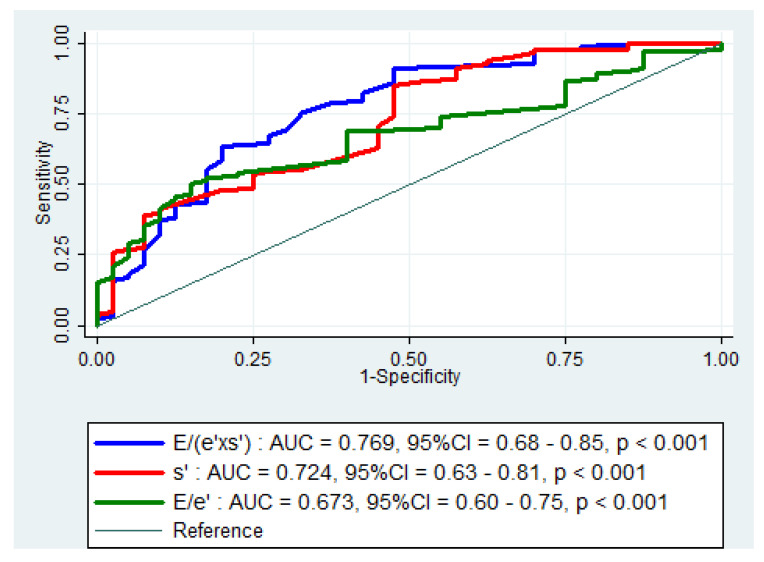
Receiver operating characteristic (ROC) curves for E/(e’×s’), E/e’ ratio, and s’ velocity to predict cardiac events in patients with non-ST-segment elevated acute coronary syndrome. AUC = area under the ROC curve; CI = confidence interval; E = peak early diastolic transmitral velocity; e’ = peak early diastolic mitral annular velocity; s’ = peak systolic mitral annular velocity.

Figure 1 shows the ROC curves of the best echocardiographic parameters to predict cardiac events. The areas under ROC curve (AUC) identified the highest accuracy for E/(e’×s’) index (AUC = 0.769, 95%CI = 0.682–0.855, *p* < 0.001). The baseline s’ and E/e’ ratio were also significant for predicting composite outcomes (AUC = 0.724, 95%CI = 0.637–0.812, *p* < 0.001 and AUC = 0.673, 95%CI = 0.596–0.749, *p* < 0.001, respectively). A statistical comparison of the ROC curves demonstrates significant differences between E/(e’×s’) and s’ (*p* = 0.009) and between E/(e’×s’) and E/e’ (*p* = 0.003). All the other analyzed echocardiographic parameters presented a lower AUC. The optimal cut-off value for E/(e’×s’) at discharge to predict the composite outcome was 1.63 (74% sensitivity and 67% specificity).

### 3.3. Predictors of Outcome

The echocardiographic variables that predicted cardiac events on univariate Cox regression analysis (*p* < 0.05) are shown in (Table 2): LVEF, LAV, LAVI, SPAP, E, E/A, s’, E/e’, and E/(e’×s’). Conversely, LV end-diastolic volume index, LV end-systolic volume index, E-deceleration time, A, e’, RV, and ROA were not significantly associated with cardiac events on univariate analysis. Afterwards, univariate significant predictors were entered into a multivariate analysis to observe the occurrence of the cardiac events. E/(e’×s’) before discharge resulted as the single independent echocardiographic predictor of composite outcome (HR = 2.621, 95%CI = 1.308–5.252, *p* = 0.007).

After adjustments in Model 1 and Model 2 and after adjusting for all the echocardiographic measures (Model 3), the E/(e’×s’) ratio remained the only independently parameter associated with the composite outcomes and hospital readmission, respectively. Other echocardiographic measures were not associated with cardiac events and readmission for HF and/or re-infarction (Table 3). However, no echocardiographic parameter determined before hospital discharge was independently associated with cardiac death.

Model 1 is adjusted for age, gender, mean blood pressure, heart rate, body mass index, prior coronary artery disease, family history of cardiovascular disease, diabetes mellitus, current smoker, hypercholesterolemia, and hypertension.

Model 2 is adjusted for previous mentioned variables in Model 1 as well as laboratory data (N-terminal pro-brain natriuretic peptide level, peak high sensitive cardiac troponin I level), coronary angiography (culprit lesions, multivessel disease), and acute coronary syndrome category.

Model 3 is adjusted for all previous mentioned variables in Models 1 and 2, additionally left ventricular ejection fraction, systolic pulmonary artery pressure, left atrial volume, indexed left atrial volume, E velocity, E/A ratio, and e’ velocity.

### 3.4. Incremental Prognostic Yield of E/(e’×s’) Determined before Hospital Discharge to Predict Composite Outcome

Harrell c-statistics revealed that the E/(e’×s’) index contributed with a higher c-statistics compared with s’ velocity (0.721 vs. 0.708, *p* = 0.027), and along with E/e’ provided the highest c-statistics among all baseline predictors (0.721 for E/(e’×s’) vs. 0.679 for E/e’, *p* = 0.009). Adding E/(e’×s’) to all the variables included in Model 1 resulted in a significant increase in the c-statistics than Model 1 alone (Model 1 with E/(e’×s’): 0.730 (0.69–0.77) vs. 0.689 (0.65–0.72), *p* = 0.034). Model 2 significantly improved when adding E/(e’×s’) ratio: 0.741 (0.70–0.78) with E/(e’×s’) vs. 0.722 (0.68–0.76) without E/(e’×s’), *p* = 0.041. Adding E/(e’×s’) to the variables included in Model 3 displayed a significant increase in the c-statistics when compared to Model 3 alone (0.761 (0.72–0.80) vs. 0.743 (0.70–0.77), *p* = 0.047). When E/(e’×s’) was already included in the models, we identified no significant changes in Harrell’s c-statistics by adding all the analyzed echocardiographic parameters.

### 3.5. Worsening of E/(e’×s’) Ratio during Follow-Up

**Figure 2:**
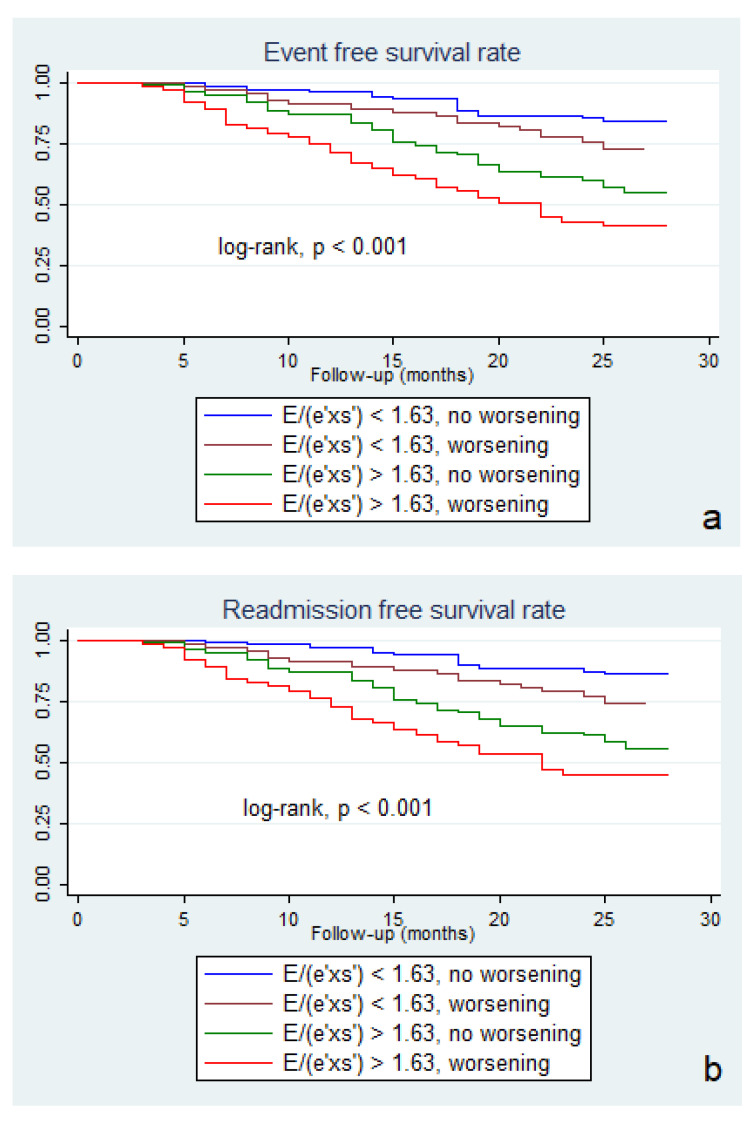

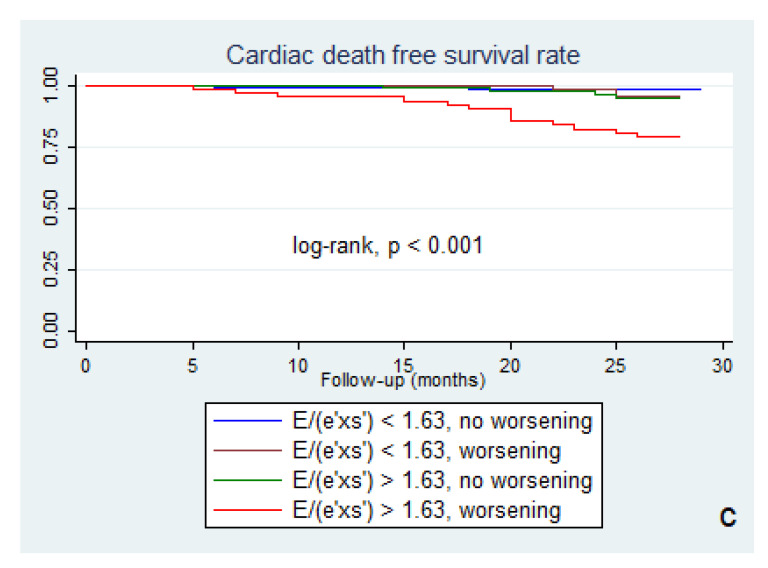
Kaplan–Meier survival curves of composite outcome (**a**), hospital re-admission (**b**) and cardiac death (**c**) according to the initial E/(e’×s’) value below and above 1.63, and to E/(e’×s’) worsening six weeks after hospital discharge. E = peak early diastolic transmitral velocity; e’ = peak early mitral annular diastolic velocity; s’ = peak systolic mitral annular.

Six weeks after hospital discharge, we identified worsening of E/(e’×s’) ratio in 140 patients (45.6%). Of these patients, 63 (20.5%) presented the initial value of E/(e’×s’) greater than 1.63. However, as shown in Figure 2, E/(e’×s’) worsening was associated with lower event-free survival rate, regardless of the E/(e’×s’) value at inclusion in the study (41.2% versus 54.5% in patients with the initial E/(e’×s’) > 1.63, and 72.7% vs. 84.1%, in those with E/(e’×s’) ≤ 1.63 at hospital discharge, respectively, log-rank, *p* = 0.001). The subgroup of patients with an initial E/(e’×s’) ratio >1.63 and worsening after six weeks presented the worst prognosis regarding composite outcome (Figure 2a), hospital readmission (Figure 2b), and cardiac death (Figure 2c), respectively.

### 3.6. Reproducibility

In 30 patients with ischemic heart disease, Bland–Altman analysis demonstrated the intra- and inter-observer agreements were good for E/(e’×s’), E/e’, and s’ measurements. The intraclass correlation coefficient for inter-observer and intra-observer variability was 0.90 and 0.89 for E/(e’×s’); 0.92 and 0.90 for E/e’; and 0.93 and 0.92 for s’, respectively.

## 4. Discussion

To the best of our knowledge, this is the first study investigating the value of E/(e’×s’) ratio to predict cardiac events (cardiac death or hospital readmission for HF and/or re-infarction) in patients with NSTE-ACS who underwent successful PCI. E/(e’×s’) provided superior prognostic information when added to all other analyzed predictors in the current population. Patients with an initial E/(e’×s’) > 1.63 and worsening after six weeks presented the worst prognosis regarding composite outcome, hospital readmission, and cardiac death.

The clinical importance of predicting prognosis in patients with NSTE-ACS has been increasing. In patients with NSTE-ACS, the rapid recognition of high-risk patients who may benefit from an intensified treatment strategy, risk stratification improves effectiveness of care to those in highest need [1,4,12]. In our study, differently from what is observed in the literature [2], there was no difference regarding the distribution of age, gender, previous cardiovascular disease, heart rate and mean arterial pressure, cardiovascular risk factors, or medication between the group with and without cardiac events. The prognosis of patients with NSTE-ACS is related to myocardial damage and LV filling pressure with impact on LA. LA size is essentially governed by the factors that affect diastolic LV filling. Previous studies with conventional echocardiographic imaging have suggested that LA size, LV volumes indices, and LVEF were good predictors for monitoring cardiovascular risk and guiding therapy in patients with ACS [6,16,17,18]. LV function evaluated with an echocardiogram before hospital discharge represents one of the strongest predictors of survival in ACS patients [19]. In our study, LVEF, LAV, LAVI, SPAP, and E/A ratio, as predictors of outcome on univariate analysis, were eliminated on multivariate analysis. The dependence of the mitral flow to the volemic status, LA pressure, myocardial relaxation, and age could explain the superiority of TDI parameters. On the other hand, biplanes assessment of LVEF is often load-dependent and neglects regional function [20].

Pulsed TDI is widely used and validated in the literature to analyze cardiac function, and it is characterized by easy accessibility and strong reproducibility. TDI has proven incremental prognostic value with respect to routine clinical, laboratory, and imaging information. This new technique does not require tracing of endocardial contours, unlike LV volumes and LVEF. Wang et al. demonstrated in a heterogeneous population of cardiac patients a better prognostic value for both e’ and E/e’ than for wall motion score index and LVEF [21]. An increased value for E/e′ represents a good prognostic index because a combination of high transmitral gradient (high E) on top of elevated minimum LV diastolic pressure (suggested by low e′) is associated with high LA pressure [22]. The E/e′ ratio has been proposed to be the single best Doppler predictor for evaluating LV filling pressure [8] and is a good prognostic marker of cardiac outcome after myocardial infarction [17,23]. Lin et al. have demonstrated the prognostic value of E/e’ in patients that underwent PCI after non-ST elevation acute myocardial infarction [9]. Other studies analyzing patients with ACS have showed that the E/e’ ratio correlates well to measured LA pressure [24] and is a powerful predictor of adverse events [1,4,7]. TDI analysis in patients with suspected NSTE-ACS has potential cost savings, as shown by Vijay S Gc et al. [4]. In a recent work, Fraser et al. demonstrate that abnormal LV diastolic function is missed by using the reductive index E/e’ alone, and more detailed measurements are needed in clinical trials and to guide treatment in individual patients [25].

A significant decrease of e’ and s’ velocities has been observed in patients with myocardial ischemia, even if cut-off values for maximum systolic and diastolic velocities that detect significant coronary lesion are still unclear [1,6,10]. In patients with ST-elevated acute myocardial infarction, a pattern of low global systolic performance (s’) or low global diastolic function (e’) is a paramount marker of greater risk for the cardiac events [10]. Systolic mitral annular velocity is related to the longitudinally orientated myocardial fibers located in the sub-endocardium, which is known to be most susceptible to ischemia [2]. This velocity, determined using TDI, appears to be useful in assessing the consequences of ischemia. Reduced s’ velocity was a more powerful predictor of outcome than LVEF in patients with ACS, as showed by Westholm et al., probably because it may reflect subclinical LV systolic dysfunction [1]. In these patients, kinetic energy dissipation within the LV increases linearly with the increase of LVEF, as the flow turbulence into the LV is higher [6]. These findings suggested that systolic longitudinal dysfunction is important in providing prognostic information for NSTE-ACS patients, and it might be relevant to disease pathophysiology.

LV diastolic dysfunction is usually the result of altered LV relaxation with or without reduced restoring forces (and early diastolic suction), and increased LV chamber stiffness, which increases LV filling pressures. Conceptually, it is very difficult to separate relaxation from contraction, and it can be considered together as part of a continuous cycle. The energy generated during systole is stored in myocardial collagen fibers, and following relaxation, the ventricle uncoils, creating LV suction. There seems to be a relation of proportionality between decline in contractile function and reduction in recoil, with parallel changes in the extracellular matrix [26,27].

Our group proposed several years ago a new TDI index, E/(e’×s’) that associates a marker of diastolic function (E/e’) and a parameter that explores LV systolic performance (s’). Recent studies have shown E/(e’×s’) to be a strong predictor of adverse outcomes in several cardiac diseases. We demonstrated the utility of this index to assess the LV filling pressure in a heterogeneous population of cardiac patients, regardless of LVEF [11] and to improve the prediction of cardiac outcomes in patients with HF [12]. Our data are in agreement with a recent study of Lee et al. [28] that identified in patients with ACS an increase in collagen turnover that is caused by more aggressive ventricular remodeling in those with greater LV filling pressure. Assuming that a perfusion defect results in subclinical systolic or diastolic dysfunction, measurement of e′ and s’ velocities could provide indirect information about myocardial perfusion. Kenar Tiryakioglu et al. reported that septal E/(e’×s’) values measured after the acute anterior myocardial infarction can strongly predict LV remodeling in the 6-month follow-up [13]. Unfortunately, there are no studies that have investigated the prognostic value of E/(e’×s’) in an NSTE-ACS population. In our work, we found that E/(e’×s’) determined before hospital discharge provided superior prognostic information on the risk of developing adverse cardiac events and hospital readmission compared to all other analyzed echocardiographic parameters.

In patients with acute myocardial infarction and severe LV systolic dysfunction, current guidelines recommend to repeat echocardiography 6–12 weeks after initial hospitalization and to measure for systolic function evaluation only LVEF, but not s’ wave [14,18]. We reevaluated E/(e’×s’) ratio after six weeks, which is in agreement with a recent study that observed a sharp decline in both morbidity and mortality in HF-recovered patients [29]. Our results showed that a value of E/(e’×s’) > 1.63 before hospital discharge and it worsening after six weeks is the strongest independent predictor for an increased risk of future cardiac events, hospital readmission, and cardiac death. This result may have implications for the risk stratification in patients with NSTE-ACS.

Clinical studies have clearly demonstrated that NT-proBNP and troponin levels are increased after episodes of ischemia [19,28,30]. Elevated NT-proBNP levels have been observed in patients with unstable angina and after PCI [19,28]. Very low levels of NT-proBNP are associated with a good prognosis, whereas high levels predict an unfavorable prognosis in the medium term. On the other hand, Chapman et al. noted that whilst hs-cTnI clearly provides important prognostic information, implementation of testing into practice did not improve outcomes [31]. The present study identified NT-proBNP and hs-cTnI to be inferior to E/(e’×s’) ratio in risk stratification of NSTE-ACS.

Our results should be considered in the context of several limitations. The most important limitation of this study is its small sample size. Therefore, larger studies are needed to validate our results. The use of standard echocardiographic assessment instead of more sophisticated methods (e.g., strain imaging) could be considered both a limitation and a strength of the study. The limitation is that strain imaging has proved to be more sensitive for detecting subclinical abnormalities of both systolic and diastolic function. The strength of our parameter is its ease of use, thanks to its availability in most of the modern echo machines, making it readily applicable for the bedside assessment of patients. We limited the analysis at two sites (medial and lateral mitral annulus), and we did not examine anterior and posterior velocities that might have provided additional information. We are conscious that the E/(e’×s’) ratio is a global index of cardiac function that unmasks a precocious systolic and diastolic myocardial suffering, and it cannot provide regional information about perfusion. The study center functioned as a tertiary invasive center, and therefore, the study population may not reflect a general population of patients with NSTE-ACS.

## 5. Conclusions

In summary, our findings indicate that in patients with NSTE-ACS who underwent successful PCI, E/(E’×S’) is an important independent prognostic index of cardiac events (cardiac death or hospital readmission for HF and/or re-infarction). E/(e’×s’) provided superior prognostic information when added to other clinical, laboratory, and echocardiographic predictors. Patients with an initial E/(e’×s’) > 1.63 that worsened after six weeks presented the worst prognosis regarding composite outcome, hospital readmission, and cardiac death.

## Figures and Tables

**Table 1:** Baseline characteristics of the study groups.

Characteristics	Event Free (*n* = 201)	Cardiac Events (*n* = 106)	*p*-Value
**Clinical characteristics**			
Age, years	60.9 ± 11.1	61.4 ± 12.3	0.732
Female/male gender	55/146	35/71	0.35
Body mass index, kg/m^2^	27.5 ± 4.66	26.1 ±4.33	0.011
Heart rate, beats/min	83 ± 19	89 ± 24	0.32
Mean arterial pressure, mmHg	98.8 ± 12.9	96.9 ± 14.1	0.27
Previous coronary artery disease, *n* (%)	53 (26.3)	24 (22.6)	0.49
Unstable angina, *n* (%)	140 (69.6)	42 (39.6)	0.001
Non-STEMI, *n* (%)	61 (30.4)	64 (60.4)	0.001
Hypercholesterolemia, *n* (%)	139 (69.1)	70 (66)	0.60
Current smoker, *n* (%)	165 (82)	90 (84.9)	0.63
Diabetes mellitus	94 (46.7)	48 (45.2)	0.81
Systemic hypertension, *n* (%)	74 (36.8)	44 (41.5)	0.46
Family history of cardiovascular disease, *n* (%)	47 (23.3)	26 (24.5)	0.88
**Laboratory finding**			
NTproBNP, pg/mL	840 ± 1181	2355 ± 2277	0.001
Peak high sensitivity cardiac troponin I, ng/L	39 ± 108	86 ± 158	0.006
**Culprit lesion**			
Left anterior descending, *n* (%)	58 (28.8)	28 (26.4)	0.69
Circumflex artery, *n* (%)	36 (17.9)	21 (19.8)	0.75
Right coronary artery, *n* (%)	82 (40.8)	44 (41.5)	0.90
Left main stem coronary artery, *n* (%)	25 (12.5)	13 (12.3)	1
Multivessel lesion, *n* (%)	51 (25.3)	25 (23.5)	0.78
**Therapy at hospital discharge**			
Beta blocker, *n* (%)	185 (92.0)	93 (87.7)	0.22
ACEI/angiotensin receptor antagonist, *n* (%)	124 (61.6)	68 (64.1)	0.61
Diuretics, *n* (%)	150 (74.6)	80 (75.4)	0.89
Calcium blocker, *n* (%)	47 (23.3)	26 (24.5)	0.85
Nitrates, *n* (%)	139 (69.1)	70 (66.0)	0.61
Aspirin, *n* (%)	201 (100)	106 (100)	1
P2Y12 inhibitor, *n* (%)	201 (100)	106 (100)	1
Statin	199 (99)	104 (98.1)	0.98
**Echocardiographic indices at hospital discharge**			
LV end-diastolic volume index, ml/m^2^	69 ± 19	73 ± 15	0.065
LV end-systolic volume index, ml/m^2^	38 ± 16	43 ± 17	0.045
LV ejection fraction, %	45 ± 10	41 ± 12	0.002
Left atrial volume, ml	86 ± 37	98 ± 47	0.017
Indexed left atrial volume, mL/m^2^	46 ± 21	51 ± 26	0.061
Systolic pulmonary artery pressure, mmHg	38 ± 12	43 ± 13	0.001
Mitral regurgitant orifice area, mm^2^	23 ± 14	26 ± 11	0.053
Mitral regurgitant volume, mL	36 ± 13	39 ± 16	0.061
E, cm/s	74 ± 26	85 ± 27	0.001
E/A ratio	1.05 ± 0.67	1.36 ± 0.89	0.002
E-deceleration time, ms	174 ± 72	162 ± 75	0.14
e’, cm/s	8.3 ± 3.3	7.6 ± 2.7	0.062
E/e’ ratio	10.1 ± 4.4	12 ± 4.4	<0.001
s’, cm/s	7.5 ± 2.7	6.1 ± 2.3	<0.001
E/(e’×s’) ratio	1.61 ± 1.12	2.22 ± 1.03	<0.001

A = late transmitral flow velocity; ACEI = angiotensin-converting enzyme inhibitor; E = early diastolic transmitral flow velocity; e’ = early mitral annular diastolic velocity; LV = left ventricle; Non-STEMI = non ST-elevation acute myocardial infarction; NTproBNP = N-terminal pro-brain natriuretic peptide; s’ = systolic velocity of mitral annulus.

**Table 2:** Echocardiographic variables at hospital discharge associated with composite endpoints (hospital readmission or cardiac death) in Cox univariate and multivariate analysis.

Variables	Univariate HR (CI 95%)	*p* Value	Multivariate HR (CI 95%)	*p* Value
LV end-diastolic volume index	1.020 (0.996–1.046)	0.094	NA	NA
LV end-systolic volume index	1.014 (1.002–1.030)	0.061	NA	NA
LVEF	0.977 (0.962–0.991)	0.003	0.999 (0.980–1.018)	0.947
Left atrial volume	1.005 (1.001–1.009)	0.011	1.001 (0.987–1.014)	0.863
Indexed left atrial volume	1.008 (1.001–1.015)	0.021	0.994 (0.972–1.018)	0.670
SPAP	1.023 (1.010–1.037)	0.001	1.007 (0.991–1.023)	0.312
Mitral regurgitant orifice area	1.024 (0.966–1.082)	0.108	NA	NA
Mitral regurgitant volume	1.012 (1.002–1.024)	0.052	NA	NA
E velocity	1.012 (1.006–1.019)	0.001	1.009 (0.997–1.020)	0.123
E-deceleration time	0.998 (0.995–1.001)	0.110	NA	NA
A velocity	0.995 (0.989–1.002)	0.159	NA	NA
E/A ratio	1.452 (1.194–1.767)	0.001	1.017 (0.743–1.393)	0.914
e’ velocity	0.937 (0.877–1.001)	0.055	NA	NA
E/e’ ratio	1.084 (1.044–1.125)	<0.001	0.961 (0.900–1.027)	0.248
s’ velocity	0.819 (0.748–0.897)	<0.001	0.915 (0.803–1.043)	0.186
E/(e’×s’) ratio	1.396 (1.219–1.599)	<0.001	2.621 (1.308–5.252)	0.007

AA = peak late diastolic transmitral velocity; CI = confidence interval; E = peak early diastolic transmitral velocity; e’ = peak mitral annular diastolic velocity; HR = hazard ratio; LVEF = left ventricular ejection fraction; s’ = peak systolic velocity of mitral annulus; NA = not applicable; SPAP = systolic pulmonary artery pressure.

**Table 3:** Multivariable Cox regression associated with cardiac events, hospital readmission, and cardiac death.

	Composite Endpoint (106 Events) Hazard Ratio (95% CI)	*p* Value	Hospital Readmission (99 Events) Hazard Ratio (95% CI)	*p* Value	Cardiac Deaths (22 Events) Hazard Ratio (95% CI)	*p* Value
**Model 1**						
E/(e’×s’)	3.33 (1.400–7.918)	0.006	3.75 (1.529–9.922)	0.004	3.20 (0.379–27.12)	0.285
E/e’	0.97 (0.909–1.055)	0.586	0.97 (0.902–1.051)	0.506	0.99 (0.848–1.164)	0.965
s’	0.94 (0.834–1.078)	0.419	0.96 (0.844–1.099)	0.583	0.91 (0.646–1.303)	0.631
**Model 2**						
E/(e’×s’)	3.78 (1.565–9.174)	0.001	3.72 (1.536–9.037)	0.004	2.35 (0.237–23.36)	0.464
E/e’	0.98 (0.911–1.057)	0.631	0.96 (0.894–1.044)	0.262	0.97 (0.828–1.155)	0.798
s’	0.97 (0.849–1.110)	0.674	0.98 (0.861–1.124)	0.386	0.93 (0.625–1.390)	0.732
**Model 3**						
E/(e’×s’)	3.32 (1.368–8.093)	0.008	3.39 (1.349–8.524)	0.009	2.81 (0.260–30.38)	0.394
E/e’	0.92 (0.801–1.061)	0.259	0.91 (0.792–1.059)	0.239	1.19 (0.897–1.588)	0.223
s’	0.95 (0.818–1.108)	0.531	0.97 (0.836–1.147)	0.801	0.92 (0.581–1.484)	0.759

A = peak late diastolic transmitral velocity; CI = confidence interval; E = peak early diastolic transmitral velocity; e’ = peak mitral annular diastolic velocity; HR = hazard ratio; s’ = peak systolic velocity of mitral annulus.

## Data Availability

The datasets used and analyzed during the current study are available from the corresponding author on reasonable request.
